# The Impact of Comorbidities and Intensive Care Unit (ICU) Admissions on Survival in Kyphoscoliosis-Related Respiratory Failure: A Retrospective Cohort Study

**DOI:** 10.3390/jcm14103516

**Published:** 2025-05-17

**Authors:** Eylem Tunçay, Sinem Güngör, Buse Nur Ertam, Birsen Ocaklı, Emine Aksoy, Özlem Yazıcıoğlu Moçin, Gökay Güngor, Nalan Adıgüzel, Zühal Karakurt

**Affiliations:** 1 Department of Pulmonary Diseases, University of Health Sciences, Şehit Prof Dr. İlhan Varank Training and Research Hospital, 34785 Istanbul, Türkiye; 2 Intensive Care Unit, University of Health Sciences, Sureyyapasa Pulmonary Disease and Pulmonary Surgery Training and Research Hospital, 34844 Istanbul, Türkiye; sinemgungor@hotmail.com (S.G.); birsenocakli@hotmail.com (B.O.); dremineaksoy95@gmail.com (E.A.); ozyaz@hotmail.com (Ö.Y.M.); drgokaygungor@hotmail.com (G.G.); nlnadiguzel@gmail.com (N.A.); zuhalkarakurt@hotmail.com (Z.K.); 3 Department of Pulmonary Diseases, Hatay Defne State Hospital, 31000 Defne, Türkiye; buse.ertam@hotmail.com

**Keywords:** non-invasive ventilation, coronary artery disease, kyphoscoliosis, mortality

## Abstract

**Background**: Kyphoscoliosis is a restrictive thoracic disorder frequently associated with chronic respiratory failure. While home non-invasive ventilation (NIV) improves short-term outcomes, long-term mortality predictors remain underexplored in this context. **Methods**: This retrospective cohort study evaluated 88 kyphoscoliosis patients with chronic respiratory failure who were initiated on home-based NIV between 2008 and 2018 at a tertiary ICU outpatient clinic. The demographic, clinical, and laboratory data were analyzed. Survival was assessed using Kaplan–Meier analysis, and independent predictors of mortality were identified via Cox regression. **Results**: Among the 88 patients (52% male), 28 (32%) died during long-term follow-up. Age, BMI, pulmonary function, and arterial blood gas values were similar between survivors and non-survivors. Non-survivors had significantly higher mMRC dyspnea scores, were more likely to be active smokers, and had a higher prevalence of coronary artery disease (CAD) (*p* = 0.015). The Kaplan–Meier survival analysis revealed significantly lower survival in patients with CAD (*p* = 0.021) and active smokers (*p* = 0.034). Cox regression analysis indicated that the presence of CAD (HR: 5.69, 95% CI: 1.34–24.08, *p* = 0.018) and hospital admission after the initiation of home-based NIV therapy (HR: 1.97, 95% CI: 1.01–3.85, *p* = 0.040) increased the risk of mortality. Conversely, a higher pH at the last outpatient visit was associated with improved survival (HR: 0.50, 95% CI: 0.00–0.692, *p* = 0.003). **Conclusions**: CAD, pH value, and increased ICU admissions after the initiation of home-based NIV are predictors of mortality in kyphoscoliosis patients. The study results highlight reduced survival associated with the presence of coronary artery disease and smoking, emphasizing the importance of routine cardiovascular assessment and close clinical follow-up in this high-risk population.

## 1. Introduction

Kyphoscoliosis is a condition that arises from respiratory muscle weakness due to the deterioration of the chest wall’s stability and respiratory mechanics. It may develop due to various causes, including childhood trauma, spinal tuberculosis, post-rachitic changes, and neuromuscular disorders [1]. Non-invasive mechanical ventilation (NIV) is a widely accepted treatment for chronic respiratory failure due to kyphoscoliosis [2,3].

In kyphoscoliosis patients, home use of non-invasive ventilation (NIV) has been shown to reduce exacerbations and hospital admissions while improving the daytime PaCO_2_ and PaO_2_ levels [3,4]. Furthermore, NIV therapy increased survival threefold compared with long-term oxygen therapy [5].

Although the short-term benefits of home non-invasive ventilation use in chest wall diseases with chronic respiratory failure are well known, there are few studies on its long-term effects, especially in small patient groups [6,7,8].

In a study investigating the long-term follow-up outcomes of patients with tuberculosis sequelae and kyphoscoliosis, no separate analysis was performed specifically for kyphoscoliosis; however, female sex, younger age, higher body mass index, and oxygen and carbon dioxide levels were identified as prognostic indicators associated with improved survival [6].

An evaluation of patients with tuberculosis sequelae and kyphoscoliosis who received non-invasive ventilation for chronic stable respiratory failure identified hypercapnia (PaCO_2_ > 50 mmHg) and multiple comorbidities (Charlson comorbidity index > 3) as risk factors for mortality over a 10-year follow-up period [7].

There is a lack of studies examining the risk factors associated with mortality in the long-term follow-up of patients with kyphoscoliosis undergoing non-invasive ventilation for chronic respiratory failure, while also taking disease progression into consideration. In our study, we aimed to investigate the factors affecting mortality in patients with kyphoscoliosis using non-invasive ventilation at home due to chronic respiratory failure during their long-term follow-up.

## 2. Methods

This retrospective observational cohort study included patients with kyphoscoliosis who were initiated on home-based non-invasive ventilation due to chronic respiratory failure. All kyphoscoliosis patients with CRF followed up between 1 January 2008 and 31 December 2018 in the intensive care unit (ICU) outpatient clinic of a tertiary care reference hospital specializing in pulmonary diseases were included.

This study was conducted in accordance with the World Medical Association Declaration of Helsinki—Ethical Principles for Medical Research Involving Human Subjects. Ethical approval for this study, including the review and analysis of historical patient records, was obtained from the Ethics Committee of the University of Health Sciences, Şehit Prof. Dr. İlhan Varank Training and Research Hospital (Approval Date/Number: 12 February 2025/028). Due to the retrospective design of this study, obtaining informed consent from individual patients was not feasible. However, all the data were fully anonymized prior to analysis to ensure patient confidentiality.

### 2.1. Chronic Respiratory Failure (CRF) Outpatient Clinic Setting

The “CRF outpatient clinic” was established to monitor patients receiving long-term oxygen therapy, non-invasive ventilation, and invasive mechanical ventilation due to various conditions, including kyphoscoliosis, obesity hypoventilation syndrome, obstructive sleep apnea, neuromuscular disorders, bronchiectasis, COPD, asthma, and sequelae of tuberculosis, as well as patients with tracheostomies. Routine follow-ups were conducted approximately 1, 3, and 6 months after ICU discharge. In addition, the outpatient clinic also documented the number of exacerbations and emergency unit, hospital, and ICU admissions in the year prior to the initiation of home-based NIV therapy [9].

**Figure 1:**
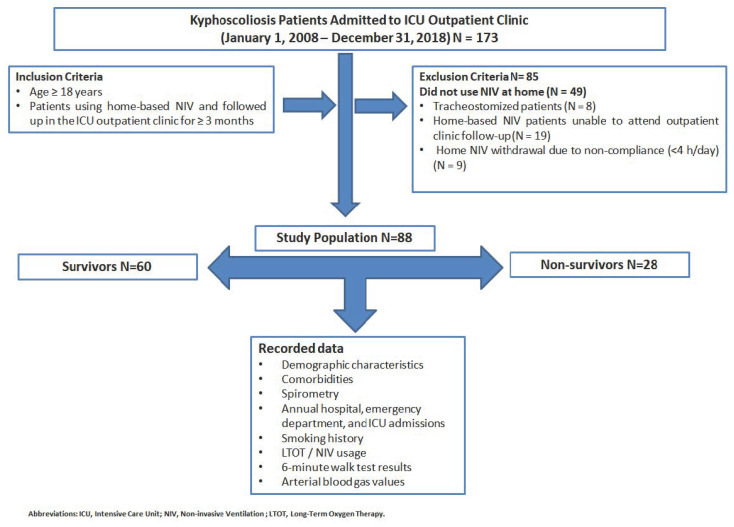
Flowchart of this study.

The study flowchart is presented in Figure 1.

### 2.2. Patients

The inclusion criteria were as follows:

- Age ≥ 18 years;
- Patients using home-based NIV followed up in the ICU outpatient clinic ≥ 3 months.

The exclusion criteria were as follows:

- Patients discharged from the ICU without NIV;
- Tracheostomized patients;
- Patients undergoing home-based NIV who were unable to attend outpatient clinic follow-up;
- Home-based NIV withdrawal due to non-compliance (<4 h/day).

Data were collected from the hospital information system and outpatient clinic records, including demographic characteristics, diagnoses, body mass index (BMI), smoking history, comorbidities, pulmonary function test results (forced expiratory volume in the first second [FEV1], forced vital capacity [FVC], forced expiratory volume in the first second/forced vital capacity [FEV1/FVC]), arterial blood gas (ABG) values, Modified Medical Research Council (mMRC) score, Charlson comorbidity index score, duration and type of NIV use, number of hospital admissions (ward, ICU) before and after NIV therapy, and mortality during follow-up.

### 2.3. Definitions

#### 2.3.1. Chronic Respiratory Failure

Chronic respiratory failure was considered as chronic hypoxia (PaO_2_ < 55 mmHg) and/or chronic hypercarbia (PaCO_2_ > 45 mmHg) in ABG examined during the stable period in restrictive lung diseases [10].

#### 2.3.2. Restrictive Lung Diseases (Kyphoscoliosis)

Chronic respiratory failure due to kyphoscoliosis was classified as restrictive lung disease.

#### 2.3.3. Indications for NIV Use at Home in Kyphoscoliosis Patients

Patients with chest wall deformity and symptoms such as fatigue, dyspnea, and morning headache should be evaluated for home-based NIV as supportive therapy in the case of hypoventilation and hypercarbia (PaCO_2_ > 45 mmHg) during the day/wakefulness period [11].

NIV treatment at home is indicated in cases of symptoms secondary to hypercarbia and nocturnal hypoventilation in patients with chronic respiratory failure, as well as in cases in which respiratory failure cannot be managed during the stable period despite adequate medical and oxygen treatments [12].

### 2.4. Statistical Analysis

The SPSS Windows Version 20.0 software was used for statistical analyses. Descriptive statistics were calculated to analyze the demographic and clinical data of the patients. The normality of the data was tested using the Kolmogorov–Smirnov test (*p* > 0.05 was considered a normal distribution, whereas *p* < 0.05 was considered non-parametric). Student’s *t*-test was used for parametric analyses, and values are expressed as the mean and standard deviation (SD) for continuous variables with a normal distribution. The Mann–Whitney U test was used for non-normally distributed continuous variables, and the associated values are expressed as the median and interquartile range (IQR). Pearson’s chi-squared and Fisher’s exact tests were used to analyze the relationships between categorical variables. Cox regression analysis was performed for mortality risk analysis during the follow-up period. The Kaplan–Meier curve and log-rank test were used for survival analysis. The results were evaluated using a 95% confidence interval (CI). A *p*-value < 0.05 was considered statistically significant.

## 3. Results

A total of 88 patients diagnosed with chronic respiratory failure due to kyphoscoliosis and receiving domiciliary non-invasive mechanical ventilation (NIV) were included in this retrospective study. Of these patients, 46 (52%) were male, and 28 (32%) died during the follow-up period. Patients were classified into two groups: survivors (*n* = 60, 68%) and non-survivors (*n* = 28, 32%), and their demographic and clinical characteristics were compared.

There were no significant differences between survivors and non-survivors in terms of gender (*p* = 0.53) and median age (60 years [49–69] vs. 62 years [52–73], *p* = 0.54). Similarly, body mass index (BMI), arterial blood gas (ABG) values, and pulmonary function test (PFT) results did not significantly differ between the two groups (all *p* > 0.05); see Table 1.

The prevalence of comorbidities differed significantly across smoking status groups (*p* = 0.027). Comorbid conditions were most frequent in non-smokers (28, 59.6%), followed by smokers (4, 8.5%) and ex-smokers (15, 31.9%). Although the difference in coronary artery disease (CAD) prevalence among the groups was not statistically significant (*p* = 0.724), the rate was higher in non-smokers (9, 50%) than in ex-smokers (6, 33%) and smokers (3, 16.7%).

Non-survivors had significantly higher mMRC scores, indicating worse dyspnea (*p* < 0.001). Active smoking was more prevalent in non-survivors than in survivors (32% vs. 11%, *p* = 0.049), and the prevalence of coronary artery disease (CAD) was significantly higher in non-survivors (35.7% vs. 8%, *p* = 0.015). Non-survivors had significantly higher pre-NIV hospital admissions per year (median: 2 vs. 1, *p* = 0.002) and required more intensive care unit (ICU) admissions after NIV initiation (*p* < 0.001). These results are summarized in Table 1.

**Figure 2:**
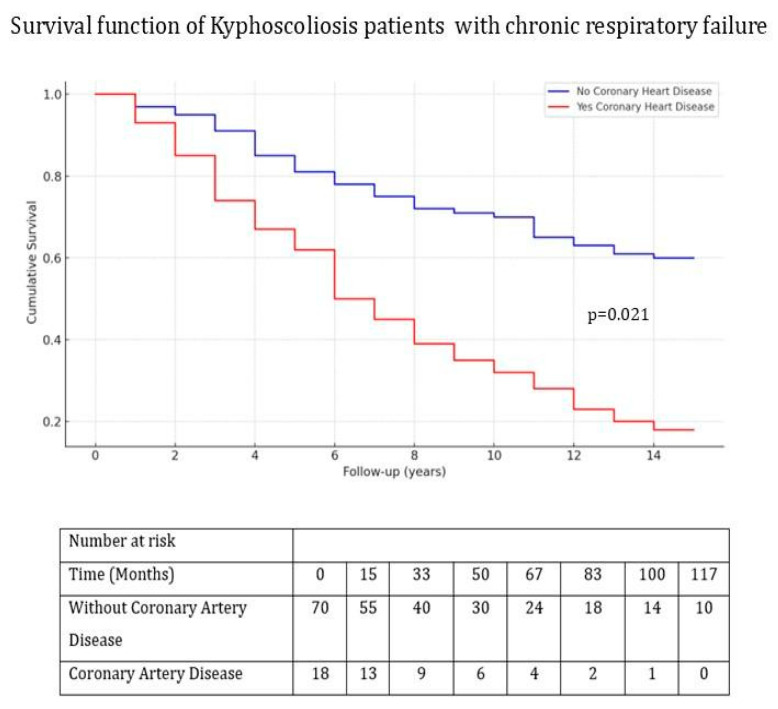
Kaplan–Meier survival curves comparing patients with and without coronary artery disease (CAD). The log-rank test demonstrated a statistically significant difference between groups (*p* = 0.021). The number of patients at risk during each follow-up time point (in months) is shown below the *x*-axis.

**Figure 3:**
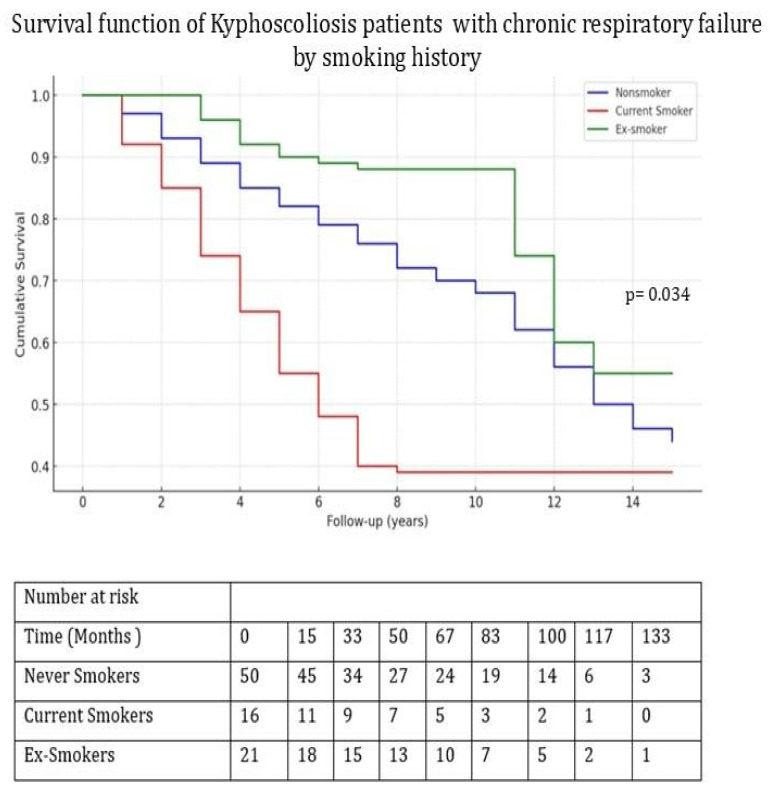
Kaplan–Meier survival curves comparing never smokers, current smokers, and ex-smokers. The log-rank test indicated a statistically significant difference between groups (*p* = 0.034). The number of patients at risk during each follow-up time point (in months) is shown below the x-axis.

The median total follow-up period was significantly shorter in non-survivors (1144 days [910–2336] vs. 2499 days [1430–3586], *p* = 0.003). Kaplan–Meier survival analysis was conducted, and a log-rank test was performed to evaluate the impacts of cardiovascular disease and smoking status on survival outcomes. Survival rates were significantly lower in patients with CAD (*p* = 0.021) and in active smokers (*p* = 0.034); see Figure 2 and Figure 3.

Cox regression analysis was performed to identify the independent predictors of mortality in patients with kyphoscoliosis receiving NIV. A history of coronary artery disease (CAD) was associated with a 5.69-fold increased risk of mortality (HR: 5.69, 95% CI: 1.34–24.08, *p* = 0.018), and a higher frequency of post-NIV ICU admissions was linked to a 1.97-fold increase in mortality risk (HR: 1.97, 95% CI: 1.01–3.85, *p* = 0.040). Conversely, higher pH values at the final outpatient visit were associated with a 50% reduction in mortality risk (HR: 0.50, 95% CI: 0.01–0.69, *p* = 0.003). Neither an mMRC score ≥ 2 (HR: 2.09, 95% CI: 0.27–16.31, *p* = 0.581) nor the smoking status (HR: 1.22, 95% CI: 0.52–2.91, *p* = 0.648) were found to be significant predictors of mortality (Table 2).

## 4. Discussion

In our study investigating risk factors associated with long-term mortality in patients with chronic respiratory failure due to kyphoscoliosis receiving home-based NIV, the survivor group presented lower mMRC dyspnea scores. Survival rates were lower among patients with coronary artery disease and active smokers. Increased post-NIV ICU admissions and a history of coronary artery disease were associated with a higher risk of mortality, while higher pH levels at the final outpatient visit correlated with better survival outcomes.

To date, no adequate data examining the relationship between the mMRC dyspnea score and mortality in patients with chronic respiratory failure due to scoliosis receiving home-based NIV have been provided. However, increased dyspnea severity is generally associated with a higher mortality risk in chronic respiratory failure. It has been reported that a significant association between the mMRC dyspnea scale and mortality was detected in patients with CRF receiving NIV therapy [13,14].

In patients with non-paralytic kyphoscoliosis, survival was found to be better in those using home-based NIV alone than that in those using long-term oxygen therapy in addition to NIV [5].

Niccolini et al. demonstrated that, in a univariate analysis of 18 kyphoscoliosis patients who were followed for four years with home mouthpiece ventilation and NIV after hospitalization for acute respiratory failure, the presence of cardiac comorbidity; low pH, MIP, and SNIP values; high respiratory rate; and elevated PaCO_2_ and oxygen desaturation index (ODI) at admission were associated with mortality. In their study, multivariate analysis was not performed due to the small sample size; the sleep data were limited, and the clinical and functional assessments were only conducted at the 6-month follow-up during the 4-year observation period [15]. Marti et al. evaluated 110 patients with chest wall deformities (CWDs)—61 with sequelae of tuberculosis and 49 with kyphoscoliosis—and found that a Charlson comorbidity index ≥ 3 was associated with a 6.6-fold increase in the risk of mortality [7]. In the study by Adıgüzel et al., while cor pulmonale was one of the leading causes of ICU admission, long-term mortality risk factors could not be investigated due to the small sample size (*n* = 32) of the study population [8]. The current study demonstrated that kyphoscoliosis patients with concomitant coronary artery disease had significantly lower survival rates, with the presence of coronary artery disease being associated with a markedly increased risk of mortality. Notably, our study was limited to patients with kyphoscoliosis and did not encompass the entire spectrum of chest wall deformities.

In a study conducted by Marti et al., airway obstruction with restrictive respiratory pattern was observed in patients with CWD. While the researchers attributed some of the accompanying airway obstruction to tuberculosis sequelae, smoking was shown to be the reason in those with kyphoscoliosis, which accounted for 56%. However, smoking and airway obstruction were not determined as risk factors for mortality in the multivariate analysis [7]. In the present study, although active smoking was associated with decreased survival in the univariate Kaplan–Meier analysis, it did not remain a significant independent predictor in the multivariate Cox regression model. This discrepancy may be explained by the limited sample size and number of events such as mortality, which could reduce the statistical power for the detection of weaker associations in multivariate models. The higher survival observed in ex-smokers compared with non-smokers may be attributed to the lower prevalence of comorbidities in the ex-smoker group. Moreover, although not statistically significant, the prevalence of coronary artery disease was also lower among ex-smokers, which might have contributed to their improved survival outcomes.

Tsuboi et al. demonstrated that a low PaCO_2_ level was associated with better device compliance in patients with restrictive lung disease due to tuberculosis sequelae who were using home non-invasive ventilation at 3 to 6 months post-discharge [16]. Similarly, Martí et al. reported that a PaCO_2_ level ≥ 50 mmHg at 1 month increased mortality by 3.4 times in patients with kyphoscoliosis receiving home-based NIV [7]. In our study, no association was found between PaCO_2_ levels and mortality. Consistently, a decrease in the pH level was correlated with a 50% increase in mortality risk.

Leger et al. demonstrated that the addition of NIV to the treatment in patients with kyphoscoliosis and post-tuberculosis sequelae significantly reduced the number of hospital re-admissions due to exacerbations [17]. The association between the frequency of ICU admission after the initiation of home-based NIV therapy and mortality in kyphoscoliosis patients using long-term home-based NIV has not been extensively studied previously. However, the present study identified the number of ICU admissions as a significant predictor of mortality; in particular, a higher frequency of post-NIV ICU admissions was associated with a 1.97-fold increase in the risk of mortality.

The application of NIV in kyphoscoliosis patients is known to improve the quality and duration of REM sleep, enhance oxygenation, and reduce PaCO_2_; however, it has not been shown to affect spirometric values [18]. In our study, although its impact on spirometry was not directly evaluated, no difference was found in the spirometry values between the survivor and non-survivor groups.

This study has certain limitations. First, it was conducted in a single center, which may limit the generalizability of the obtained findings to the broader population. However, the fact that all patients were followed up in the ICU outpatient clinic by the same clinical team and under a standardized protocol enhances the internal consistency and may provide valuable insights for similar patient populations. Second, due to the retrospective nature of this study, some patient data were incomplete. As a result, individuals with missing follow-up information were excluded from the analysis. As previous studies included relatively small populations, only a few were able to perform multivariate analyses to explore mortality risk factors. In the present study, both Cox regression and Kaplan–Meier survival analyses were conducted, thus providing a more comprehensive evaluation.

## 5. Conclusions

This study demonstrated that coronary artery disease and frequent ICU admissions following NIV are significantly associated with long-term mortality in patients with kyphoscoliosis receiving home-based NIV for chronic respiratory failure. In contrast, a higher pH value at the final outpatient visit was associated with improved survival, highlighting the importance of close metabolic monitoring in this patient group. Although the mMRC dyspnea score and smoking status were associated with reduced survival in univariate analysis, their independent associations did not emerge in the multivariate model. These findings emphasize the necessity of a comprehensive cardiovascular evaluation following the initiation of home-based NIV. Furthermore, based on our findings, regular follow-up by a multidisciplinary team—including arterial blood gas analysis, assessment of device compliance, and monitoring of hospital or ICU admissions—is suggested for kyphoscoliosis patients receiving home-based NIV.

## Figures and Tables

**Table 1:** Comparison of demographic features, ABG, pulmonary function test values, and hospital admission data of Kyphoscoliosis patients with chronic respiratory failure receiving NIV.

	Survivors *n* = 60	Non-Survivors *n* = 28	*p*-Value
Age, median (IQR)	60 (49–69)	62 (52–73)	0.54
Male, *n* (%)	30 (50)	16 (57)	0.53
BMI, median (IQR) (kg/m^2^)	23 (20–26)	22 (18–31)	0.79
Biomass, *n* (%)	6 (11)	5 (18)	0.4
Smoking, *n* (%)			
Non-smoker	35 (59)	15 (54)	**0.04**
Smoker	7 (12)	9 (32)	
Ex-smoker	17 (29)	4 (14)	
mMRC score, *n* (%)			
mMRC0, *n* (%)	0 (0)	1 (4)	**<0.001**
mMRC1, *n* (%)	8 (14)	0 (0)	
mMRC2, *n* (%)	26 (47)	1 (4)	
mMRC3, *n* (%)	14 (25)	21 (75)	
mMRC4, *n* (%)	8 (14)	5 (17)	
Daily NIV usage time in hour, median (IQR)	5 (4–7)	6 (4–8)	0.08
Comorbidities, *n* (%)	33 (55)	14 (50)	0.66
Hypertension	19 (32)	7 (25)	0.52
CAD	8 (13)	10 (36)	**0.015**
DM	4 (7)	3 (11)	0.51
AF	5 (8)	4 (13)	0.39
Malignancy	2 (3)	1 (4)	0.95
Hospital admissions within 1 year before home-based NIV, median (IQR)	1 (1–2)	2 (1–3)	**0.002**
ICU admissions within 1 year before home-based NIV, median (IQR)	1 (1–1)	1 (1–1)	0.53
Hospital admission after initiation of home-based NIV therapy in a year, median (IQR)	0 (0–0)	0 (0–1)	**0.04**
Total hospital admission after initiation of home-based NIV therapy, median (IQR)	0 (0–0)	0 (0–0)	0.4
ICU admission after initiation of home-based NIV therapy in a year, median (IQR)	0 (0–0)	0 (0–1)	**<0.001**
Total ICU admission after initiation of home-based NIV therapy, median (IQR)	0 (0–0)	1 (0–1)	**0.002**
ABG values (first outpatient clinic visit after the initiation of home-based NIV), median (IQR)			
pH, median (IQR)	7.42 (7.38–7.45)	7.41 (7.38–7.42)	0.15
PaCO_2_, median (IQR)	48 (44–53)	48.5 (46–54)	0.48
PaO_2_, median (IQR)	65 (58–74)	68.5 (62–75)	0.21
BE, median (IQR)	5.4 (3.9–8.6)	6.5 (3.7–7.7)	1.0
SaO_2_, median (IQR)	93 (90–96)	94 (92–95)	0.79
ABG values (at the last outpatient clinic visit), median (IQR)			
pH, median (IQR)	**7.40 (7.37–7.43)**	**7.49 (7.45–7.52)**	**0.018**
PaCO_2_, median (IQR)	49 (45–52)	52 (43–59)	0.30
PaO_2_, median (IQR)	66 (60–74)	66 (62–82)	0.61
Base excess, median (IQR)	5.4	6.5 (2.6–9)	0.70
SaO_2_, median (IQR)	94 (91–95)	94 (90–97)	0.88
Pulmonary function test (first outpatient clinic visit after the initiation of home-based NIV), median (IQR)			
FEV1 mL first year, median (IQR)	600 (460–810)	715 (460–870)	0.45
FEV1% first year, median (IQR)	31 (22–37)	36 (28–46)	0.69
FVC mL first year, median (IQR)	685 (530–960)	825 (575–1068)	0.42
FVC % first year, median (IQR)	30 (21–35)	36 (28–42)	0.83
FEV1/FVC % first year, median (IQR)	79 (77–85)	84 (77–91)	0.45
Pulmonary function test (at last outpatient clinic visit), median (IQR)			
FEV1 mL last year, median (IQR)	550 (430–690)	640 (500–1010)	0.29
FEV1% last year, median (IQR)	28 (23–36)	30 (29–60)	0.29
FVC mL last year, median (IQR)	630 (530–810)	650 (610–900)	0.53
FVC % last year, median (IQR)	28 (21–37)	33 (23–55)	0.32
FEV1/FVC % last year, median (IQR)	78 (75–86)	82 (80–86)	0.09
6MWT at first outpatient clinic visit, median (IQR)	371 (288–441)	357 (245–455)	0.68
6MWT at last outpatient clinic visit, median (IQR)	444 (345–489)	426 (372–480)	0.79
Survival days, median (IQR)	2499 (1430–3586)	1144 (910–2336)	**0.003**
Follow-up days, median (IQR)	1975 (863–2726)	679 (98–1615)	**0.001**

Abbreviations: ABG, arterial blood gas; BMI, body mass index; mMRC, Modified Medical Research Council; NIV, non-invasive mechanical ventilation; DM, diabetes mellitus; CAD, coronary artery disease; AF, atrial fibrillation; IQR, interquartile range; ICU, intensive care unit; PaCO_2_, partial arterial carbon dioxide pressure; PaO_2_, partial arterial oxygen pressure; BE, base excess; SaO_2_, oxygen saturation; FEV1, forced expiratory volume in 1 s; FVC, forced vital capacity; FEV1/FVC, forced expiratory volume in 1 s/forced vital capacity; 6MWT, six-minute walk test. Bold values indicate statistically significant results (*p* < 0.05).

**Table 2:** Cox regression analysis of risk factors for mortality during follow-up in kyphoscoliosis patients with chronic respiratory failure receiving NIV.

	HR	95% CI	*p*-Value
		Lower–Upper	
mMRC score ≥ 2	2.093	0.269–16.310	0.581
CAD	**5.687**	**1.343–24.076**	**0.018**
Total ICU admission after initiation of home-based NIV therapy	**1.969**	**1.007–3.849**	**0.04**
pH at the last outpatient visit	**0.501**	**0.01–0.692**	**0.003**
Smoking status	1.224	0.515–2.910	0.648

Abbreviations: mMRC, Modified Medical Research Council; HR, hazard ratio; CAD, coronary artery disease; NIV, non-invasive mechanical ventilation. Bold values indicate statistically significant results (*p* < 0.05).

## Data Availability

The data presented in this study are available on request from the corresponding author due to privacy, legal, and ethical reasons.

## Abbreviations

The following abbreviations are used in this manuscript:

NIV	non-invasive ventilation
CAD	coronary artery disease
ICU	intensive care unit
CRF	chronic respiratory failure
BMI	body mass index
FEV1	forced expiratory volume in the first second
FVC	forced vital capacity
mMRC	Modified Medical Research Council
ODI	oxygen desaturation index
CWD	chest wall deformities
PaCO_2_	partial arterial carbon dioxide pressure
PaO_2_	partial arterial oxygen pressure
